# 

**Published:** 1889-04

**Authors:** 


PAGE

THE PATHOLOGY OF ACTINOMYCOSIS, WITH RECORD OF CASES AND EXPERIMENTS. George A. Bodamer, M.D., B.S. . . . . . 105
OSTEOLOGY OF THE CIRCUS HUDSONIUS. R. W. Shufeldt, M.D., C.M.Z.S. . . . . . 126
A CASE OF LARYNGOTOMY FOR THE CURE OF ROARING. Daniel B. Lee, M.D.V. . . . . 160
EDITORIAL DEPARTMENT . . . . . . 162
CASE DEPARTMENT . . . . . . 167
SELECTIONS FROM FOREIGN JOURNALS . . . . . . 172
PROCEEDINGS OF VETERINARY SOCIETIES . . . . . . 184
REVIEW DEPARTMENT . . . . . . 190
BOOKS AND PAMPHLETS RECEIVED . . . . . . 191

